# Micro-segmental analysis of the entry pathway and distribution of zolpidem in hair from different scalp regions after a single dose

**DOI:** 10.3389/fchem.2023.1115247

**Published:** 2023-05-05

**Authors:** Jiao-Jiao Ji, Duoqi Xu, Hui Yan, Ping Xiang, Min Shen

**Affiliations:** ^1^ Department of Forensic Toxicology, Shanghai Key Laboratory of Forensic Medicine, Academy of Forensic Science, Shanghai, China; ^2^ Department of Forensic Medicine, Shanghai Medical College, Fudan University, Shanghai, China

**Keywords:** zolpidem, single hair, micro-segmental analysis, incorporation, entry pathway, different sampling region

## Abstract

**Introduction:** Hair testing is well established for the assessment of past drug exposure; however, more research is needed to understand drug incorporation mechanisms and drug entry pathways into hair.

**Method:** In this study, a micro-segmental LC–MS/MS method was used to analyze a 0.4 mm segment of hair after a single oral administration of zolpidem. Five single hairs were plucked at 1 day, 3 days, 7 days, and 28 days after administration from the vertex posterior of three subjects, and 5 single hairs were also plucked from the parietal, left temporal, and right temporal regions of the head at 28 days.

**Results and discussion:** Proximal S1 (0–0.4 mm) in hair plucked at 1 day had the highest level of zolpidem at 1.5–2.4 pg/mm; much lower concentrations (< 1 pg/mm) were detected at proximal S2–S8 (0.4–3.2 mm). The drug concentration decreased gradually in S1 for 7 days after drug intake and disappeared by 28 days, suggesting that the drug from the bloodstream initially combined with the hair follicle and then gradually moved to the hair tip as the hair grew. The zolpidem concentration–hair segment profiles exhibited a large peak (root side) and a small peak (tip side) for the four sampling times in all three subjects, indicating that drug incorporation in the hair bulb occurred mainly from the blood but probably also entered the hair through sweat and sebum. Zolpidem was also detected in all hairs from the vertex posterior in all three subjects but was not detected in 1 hair from the parietal region and 2 hairs from the left temporal region. The consistency in drug detection, drug concentration level, and peak position was better in hair from the vertex posterior than from the other three regions, indicating that the vertex posterior is a suitable sampling region for estimating drug intake.

## 1 Introduction

Zolpidem, a nonbenzodiazepine sedative–hypnotic, is mainly used for short-term treatment of insomnia, due to its strong sleep-inducing effect within 30 min after administration (Cui et al., 2013). Because of the wide range of clinical applications for zolpidem, its abuse and misuse are serious public health concerns, as it has inherent adverse effects of a high degree of tolerance and dependence (Kim et al., 2013). Zolpidem has also become one of the date-rape drugs involved in drug-facilitated crimes (DFCs), including robbery and sexual assault (Ishida et al., 2009; Rossi et al., 2014; Madry et al., 2020). The drugs used in DFC cases are usually difficult to detect in blood and urine because of the low dosages used and because they can be quickly cleared from body fluids (Villain et al., 2004). These cases frequently have a long delay between the event and the police report, and the drugs themselves possess amnesic properties, which further complicates reporting. For these reasons, hair is a useful forensic aid and is sometimes even the only matrix available as an analytical strategy in DFCs (Kintz, 2007). Hair provides a wider window of detection than other biological samples, such as plasma and urine, as well as valuable retrospective information regarding the history of drug exposure (Xiang et al., 2015; Yan et al., 2021). However, despite the growing application of hair tests, the exact mechanism of drug incorporation into hair and its entry routes are still debated.

Initially, a widely held belief was that drugs passively diffused into the hair from blood circulating at the base of the hair follicle. However, later research indicated that the findings could not be explained only by direct drug incorporation from the bloodstream and that a more complex model involving more pathways was needed to explain how drugs enter the hair. At present, three models for incorporation have been proposed: drugs can enter the hair through 1) active or passive diffusion from the bloodstream feeding the dermal papilla, 2) diffusion from sweat and other secretions bathing the growing or mature hair fiber, or 3) external drug from vapors or powders that diffuse into the mature hair fiber. (Pragst et al., 1998; Pötsch et al., 1997; Cone, 1996). Schräder et al. (Schräder et al., 2012) analyzed the daily ethyl glucuronide (EtG) levels in the hair of three volunteers after drinking alcohol and found that the incorporation of EtG from sweat was not the dominating route of entry into the hair shaft. Instead, EtG was mainly incorporated within the hair root. Nevertheless, the deposition of EtG from sweat can play an important role in long hair, where a larger area for deposition is provided by the long hair shaft than by shaved hair stubble. Shima et al. (2017) followed the time course of changes in drug distribution along single-strand hair after a single oral administration of zolpidem and determined that zolpidem was incorporated in two regions, mainly in the hair bulb and to a lesser extent in the upper dermis zone from sweat and sebum. This conclusion was also reflected in the study by Kamata et al. (2015), who examined hair 14 days after a single dose of methoxyphenamine.

The ambiguity of drug incorporation and entry mechanisms in hair may lead to errors in estimating drug intake when hair analysis is performed at a high time resolution (Shima et al., 2017). Schräder et al. (2011) found that negative results can be expected with high probability when examining the 3 cm proximal scalp hair segment that is routinely investigated, rather than a 1 cm proximal segment, because of dilution effects due to negative hair. Conventional segmentation methods usually divide the hair into 1–2 cm segments, but this segmentation is not useful for time resolution of drug intake at the monthly level, as these analyses only allow estimations that are accurate to within several months (Kłys et al., 2005; Günther et al., 2018; Wang et al., 2018). By contrast, micro-segmental analysis, which segments a single hair strand into 0.4 mm segments that correspond to the daily rate of hair growth, improves the time resolution of drug intake to the daily level (Kuwayama et al., 2018a; Kuwayama et al., 2018b; Kuwayama et al., 2019; Kuwayama et al., 2021). However, the proportion of hair in the anagen phase and the hair growth rate differ for different areas of the scalp; therefore, the hair collection region is also important when estimating drug intake.

The aims of the present study were to investigate the incorporation and entry pathway of zolpidem into hair and to explore the distribution of zolpidem and the consistency of the distribution pattern in single hair strands taken from different areas of the scalp.

## 2 Experiment

### 2.1 Chemicals and reagents

Myslee sleeping tablets (10 mg, containing 10 mg zolpidem tartrate/tablet) were purchased from SNAFI (Hangzhou, China). An authentic zolpidem stock (1 mg/mL, in methanol) and internal standard (IS) diazepam-d_5_ were purchased from Cerilliant (Round Rock, TX, United States). HPLC-grade acetonitrile and methanol were purchased from Sigma-Aldrich (St. Louis, MO, United States); ammonium acetate (98%) and formic acid (98%) were obtained from Fluka (Buchs, Switzerland). Deionized water was purified using a Milli-Q system (Millipore, MA, United States).

### 2.2 Drug administration experiments

**FIGURE 1:**
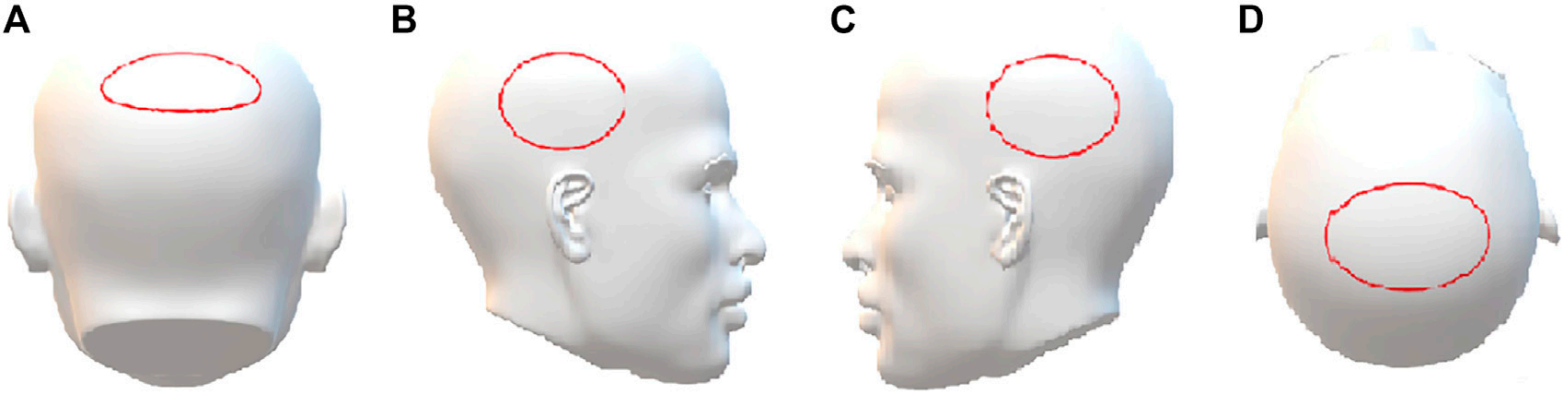
The sampling region of hair. **(A)** Vertex posterior, **(B)** left temporal, **(C)** right temporal, **(D)** parietal.

Three healthy volunteers (subjects A–C, two females and one male, all of Asian ethnicity, with straight black hair, 8 cm, 10 cm, and 10 cm in length, respectively) who had not taken any psychoactive drugs in the past year were recruited. They orally ingested one tablet (containing 10 mg zolpidem tartrate) once, and 5 single hairs (with bulb) were collected from the vertex posterior region (Figure 1A) by plucking with the roots intact using a tweezer at 1 day, 3 days, 7 days, and 28 days after the single administration (n = 5). At 28 days, 5 single hairs were also collected from the left temporal region, right temporal region, and parietal region (Figures 1B–D). The hair samples with roots were labeled and stored at room temperature until analysis. All volunteer experiments were approved by the Ethics Committee of Academy of Forensic Science, China, and written informed consent was obtained from each subject.

### 2.3 Micro-segmental analysis of single hair samples

The details of the sample preparation are described in our previous study (Xu et al., 2022), hairs were wiped with acetone to avoid external contamination that could interfere with the analysis, and each sample was allowed to dry at room temperature. Then, the length of single hair was measured and then it was cut at 0.4 mm intervals with surgical scissors while viewing under an illuminated magnifying glass (×30). Each 0.4 mm segment was placed into a 200 µL tube and 25 μL methanol containing the IS (0.5 ng/mL) was added. The samples were sonicated for 1 h and the segments were left to soak in the solution for 20 h. The supernatant was injected into the LC–MS/MS system.

The authentic 5 single hairs collected for each timepoint were analyzed individually and divided into 0.4 mm segments (S1, S2, S3….) from the root side. Considering that the segmental process is time consuming, single hair collected at different timepoints was analyzed for different length. A 0.4 cm length of hair (10 segments) was analyzed after plucking at 1 day and 3 days post administration, a 1 cm length (25 segments) was analyzed for the 7 days samples, and a 2 cm length (50 segments) was analyzed for the 28 days samples. The length and number of hairs collecting at different timepoints are shown in Table 1.

**TABLE 1:** Segment length and number of hairs collecting at different timepoints.

Collecting time	1 day	3 days	7 days	28 days
Length of analysis (cm)	0.4	0.4	1	2
Number of segments	10	10	25	50

### 2.4 Analytical method

LC–MS/MS was carried out on an ACQUITY ultra-high-performance liquid chromatograph system (Waters Corporation, United States) with an AB Sciex 6,500 plus QtrapTM triple quadrupole mass spectrometer (AB Sciex, Foster City, United States). Each 0.4 mm hair segment was separated using an Allure PFPP column (100 × 2.1 mm, 5 μm, Restek, United States) at room temperature. A gradient elution was performed using 20 mmol/L ammonium acetate with 0.1% formic acid in water (mobile phase A) and acetonitrile (mobile phase B), using the LC mobile phase gradient shown in Table 2. The autosamplers were set at 4°C, and the sample injection volume was 10 μL per injection. Molecular detection was conducted using electrospray ionization in the positive ionization (ESI^+^) mode with a multiple reaction monitoring (MRM) mode. The optimum conditions were as follows: ion spray voltage (ISV), 5500 V; source temperature (TEM), 450°C; curtain gas (CUR), 30 psi; nebulizing gas (GS1) and heater gas (GS2), both 35 psi. The transitions for zolpidem were monitored as m/z 308.1→235.1 and m/z 308.1→263.2. Analyst 1.6.3 software was used to control the system and collect the data, and MultiQuant 3.0.2 was used to analyze the data.

**TABLE 2:** The LC mobile phase gradient for zolpidem analysis.

Time (min)	Flow (mL/min)	A%	B%
Initial	0.2	95	5
1	0.2	95	5
6	0.4	10	90
7	0.4	10	90
7.5	0.4	95	5
8	0.2	95	5

Linearity for zolpidem was verified with eight different concentrations of 0.05, 0.1, 0.2, 0.5, 1, 2, 5 and 10 pg/mm with a correlation coefficient r) value above 0.99. The calibration curve was y = 0.05849x + 1.21488 fitted with 1/x weighting. The limit of detection and lower limit of quantification for zolpidem were 0.02 pg/mm and 0.05 pg/mm, respectively. Intra-day and inter-day precisions (RSD) of QC samples at three levels (0.1, 1, and 8 pg/mm) were 2.5%–7.4%. The intra-day and inter-day accuracies ranged from 96.2% to 101.4%, the extraction recoveries ranged from 98.3% to 102.1%, and the matrix effects ranged from 89.5% to 94.0%.

## 3 Results and discussion

### 3.1 entry and incorporation pathway of zolpidem into hair

The very low mass of a single 0.4-mm segment means that the exact mass cannot be determined with standard laboratory equipment. In addition, the inter-individual and intra-individual variations in the hair diameter would prevent determination of the exact concentration even when calculated based on the average weight of the hair micro-segments. Moreover, hygienic situations and weather conditions cause increasing damages and degradations of the cuticle toward the hair tip, and the water contents of the hair strand may vary significantly, an overall variation of mass of up to two–threefold seems likely between different segments. So, “mass/mass” was replaced with“mass/length” in our studies.

**FIGURE 2:**
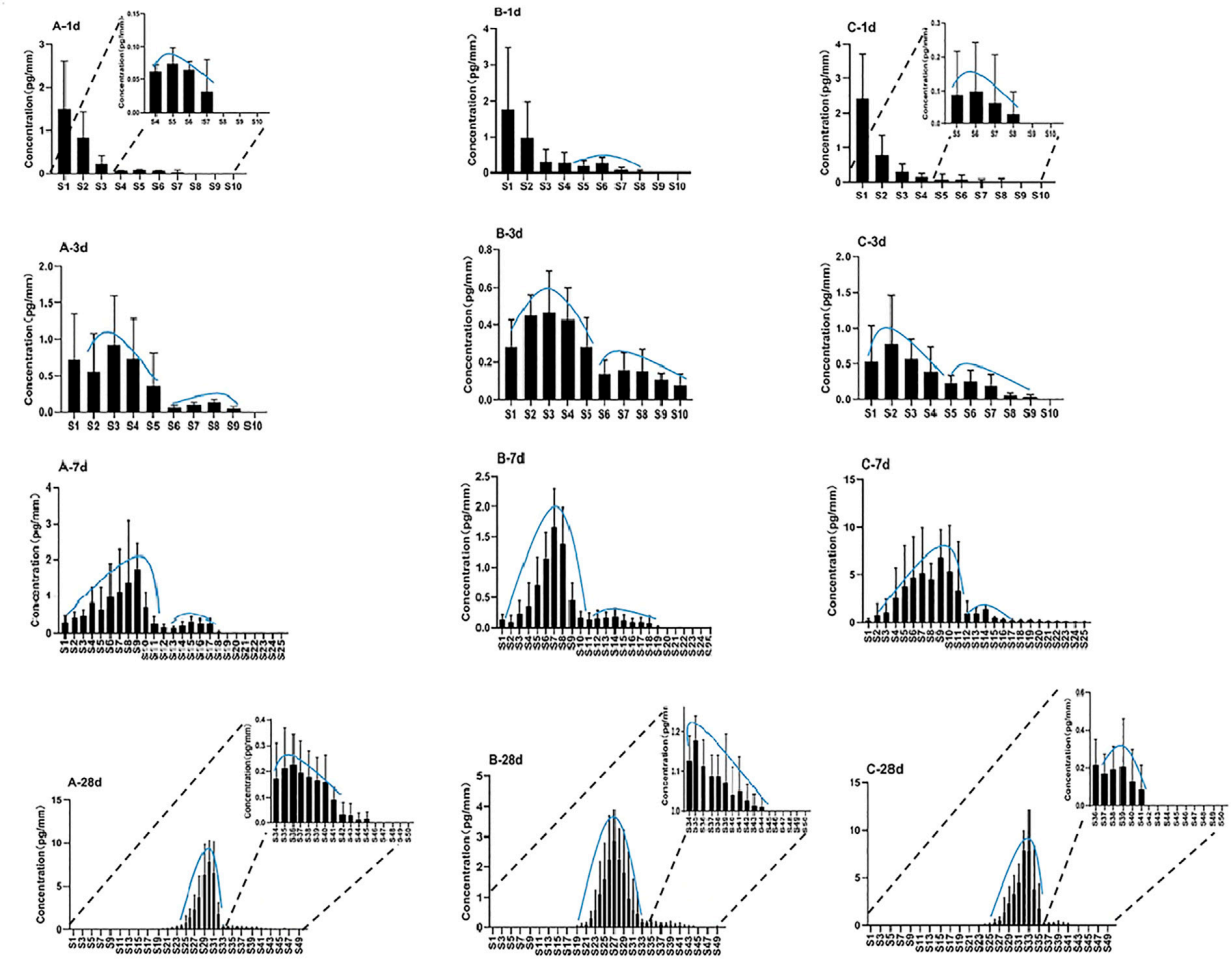
Distribution of zolpidem in single hairs at 1 day, 3 days, 7 days, 28 days after intake for subject A, B, and C.

Single hair samples collected at 1 day, 3 days, 7 days, and 28 days from the vertex posterior region of three volunteers were analyzed by LC–MS/MS. The zolpidem concentration–hair segment profiles exhibited a large peak (root side) and a small peak (tip side) for the four sampling times in all three subjects. Figure 2 shows the distribution of zolpidem in hair collected from the three subjects (n = 5) by plucking at 1 day, 3 days, 7 days, and 28 days after intake. The large peak of zolpidem moved to the hair tip as the hair grew, from S1 (0–0.4 mm from hair root) at 1 day to S32 (12.4–12.8 mm) at 28 days, and the concentration ranged from 0.5 to 8.0 pg/mm. The tip-side (small) peak concentrations of zolpidem at the four collecting times for the three subjects were between 0.07 and 1.41 pg/mm, the highest concentration was observed at 7 days after intake. The locations and concentrations of the large and small peaks for the three subjects are shown in Table 3. For the large peak, the concentration per hair for each subject gradually increased from 3 days to 28 days, suggesting that the drug accumulated in the hair shaft during this period. The weights of the proximal 2 cm of 5 single hair from the three subjects collected at 28 days after drug intake before micro-segment analysis ranged from 0.14 to 0.24 mg (mean: 0.18 mg) for subject A, 0.08–0.23 mg (mean: 0.17 mg) for subject B, and 0.14–0.45 mg (mean: 0.26 mg) for subject C. Analysis of the weight of the proximal 2 cm of a single hair and its drug concentration by one-way ANOVA revealed no significant correlation (*r* ^2^ = 0.3757, *p* = 0.29).

**TABLE 3:** Location and mean concentration of large and small zolpidem peaks in a hair strand taken from three different subjects.

Subject	Sampling time (d)	Large peak		Small peak	
		Location (from root)	Mean concentration (pg/mm)	Location (from root)	Mean concentration (pg/mm)
A	1	S1 (0–0.4 mm)	1.5	S5 (1.6–2.0 mm)	0.07
	3	S3 (0.8–1.2 mm)	0.9	S8 (2.8–3.2 mm)	0.13
	7	S9 (3.2–3.6 mm)	1.8	S15 (5.6–6 mm)	0.36
	28	S30 (11.6–12 mm)	7.8	S36 (14–14.4 mm)	0.22
B	1	S1 (0–0.4 mm)	1.7	S6 (2.0–2.4 mm)	0.24
	3	S3 (0.8–1.2 mm)	0.5	S7 (2.4–2.8 mm)	0.16
	7	S7 (2.4–2.8 mm)	1.7	S14 (5.2–5.6 mm)	0.20
	28	S27 (10.4–10.8 mm)	2.9	S35 (13.6–14 mm)	0.18
C	1	S1 (0–0.4 mm)	2.4	S6 (2.0–2.4 mm)	0.08
	3	S2 (0.4–0.8 mm)	0.8	S6 (2.0–2.4 mm)	0.25
	7	S9 (3.2–3.6 mm)	6.8	S14 (5.2–5.6 mm)	1.41
	28	S32 (12.4–12.8 mm)	8.0	S39 (15.2–15.6 mm)	0.21

At 1 day after intake, 1.5–2.4 pg/mm of zolpidem was localized in S1 (0–0.4 mm), whereas much lower concentrations (below 1 pg/mm) were commonly found between S2 (0.4–0.8 mm) and S8 (2.8–3.2 mm). The concentration in S1 of the hair samples decreased gradually at 3 days, 7 days, and 28 days after drug intake, and the concentration peak gradually moved toward the tip side. These results showed that the drug from the bloodstream initially combines with the hair follicle, and then gradually moves to the hair tip with the growth of hair. Hair follicles are embedded in the dermis of the skin and contain networks of arterial capillaries that nourish the growing hair bulb (Cone. et al., 1996) Therefore, drugs can move by passive diffusion from the bloodstream into the growing hair cells at the base of the follicle and then become tightly bound in the interior of the hair shaft during subsequent keratogenesis. Shima et al. (Shima et al., 2017) confirmed that incorporation of zolpidem from the hair bulb continued for about 2 weeks, and then the drug was successively incorporated into the hair matrix and moved toward the keratinized region as the hair grew. We also previously found much higher amounts of quetiapine and its metabolite 7-hydroxyquetiapine in guinea pig hair roots than in segmental hair shafts within the first 10 days after administration, supporting the idea that drugs enter the hair from blood circulation at the hair root (Ji et al., 2020).

The analysis of hair samples collected at 1 day after intake showed a small peak with a low concentration (below 1 pg/mm) appearing at the proximal S5 or S6 in the tip side in all three subjects. The average length of the hair root in the three subjects was 1.6 mm (about 4 segments). Therefore, the small peak was located at the upper part of the hair root. The small peak at the tip side was detected in all specimens at 3 days, 7 days, and 28 days in all three subjects, suggesting that zolpidem in the upper dermis area was incorporated within 1 day after intake through separate pathways that included the hair bulb, rather than only from the bloodstream. It was distributed over the whole hair root, but it also probably entered the hair through sweat and sebum that contained ingested zolpidem and had soaked the hair root near the scalp surface.

Our findings are consistent with previous research, as Shima et al. (2017) also showed, by quantitative sectional hair analyses of 1 mm segments of single-strand hair, that zolpidem incorporation occurred in two regions: mainly in the hair bulb from blood but to a lesser extent in the upper dermis zone from sweat and sebum. As in-depth research into drug entry pathways into hair continues, a more complex model should be used to explain the incorporation of drugs into hair. Research has suggested that drugs and metabolites in hair may also be transferred from multiple body compartments or from pools in the tissues that surround the hair follicle, including the blood during hair formation, sweat and sebum after hair formation, and from the external environment (Kamata et al., 2015). A few cases have also reported drug passage in the opposite direction, as outwardly directed transdermal migration can also occur (Pannatier et al., 1978).

### 3.2 Distribution of zolpidem in hair samples collected from different regions

**FIGURE 3:**
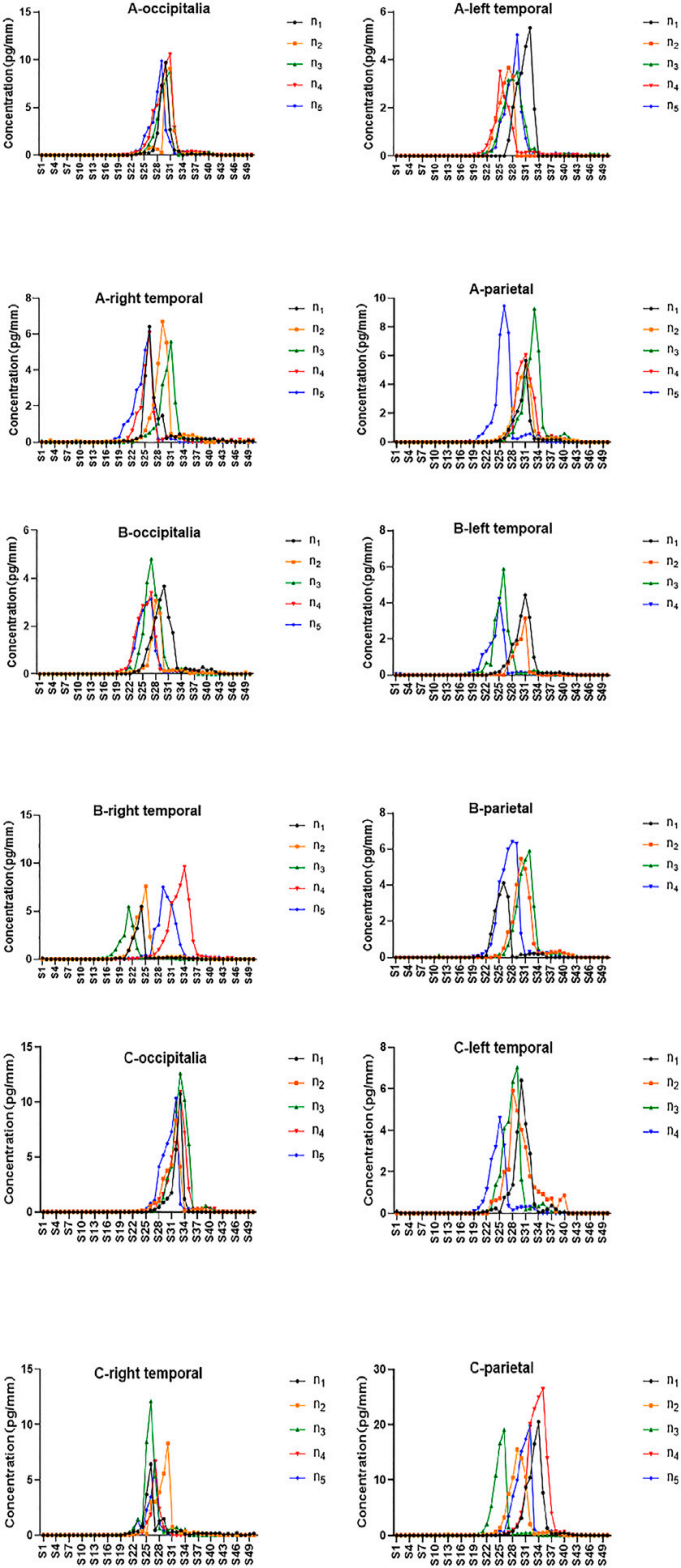
Zolpidem in single hairs for subject A, B, and C from four regions of scalp.

The drug distribution of zolpidem in hair from different regions of the head was investigated in hair collected from the vertex posterior, left temporal, right temporal, and parietal regions of the scalp. The analytical results are shown in Figure 3. Zolpidem was detected in all hairs from the vertex posterior and right temporal regions, and almost all hairs from the left temporal region (except two hairs) and the parietal region (except one hair) from all three subjects. The peak concentration of zolpidem in hair from the vertex posterior, left temporal, right temporal, and parietal regions ranged from 3.1 to 12.6 pg/mm, 3.1–7.0 pg/mm, 5.4–12.1 pg/mm, and 4.1–26 pg/mm, respectively. The peak zolpidem concentrations in hair from the vertex posterior, left temporal, right temporal, and parietal regions were located at S27–S33 (10.8–13.2 mm), S25–S32 (10–12.8 mm), S21–S34 (8.4–13.6 mm), and S26–S35 (10.4–14 mm), respectively. The locations and concentrations of the peaks in the four regions for the three subjects are shown in Table 4.

**TABLE 4:** Detection of zolpidem in single hairs taken from four different scalp regions from three subjects.

Subject	Region	Number of hairs detected	Concentration (pg/mm)	CV(%)	Location of peak	CV(%)
A	vertex posterior	5	9.6 (8.7–10.6)	7.7	S29-S31 (11.6–12.4 mm)	2.9
	left temporal	5	4.2 (3.5–5.3)	21.4	S25-S32 (10–12.8 mm)	9.2
	right temporal	5	6.2 (5.6–6.7)	6.6	S26-S31 (10.4–12.4 mm)	8.3
	parietal	5	7.2 (5.3–9.5)	28.5	S26-S33 (10.4–13.2 mm)	8.6
B	vertex posterior	5	3.6 (3.1–4.8)	19.5	S27-S30 (10.8–12 mm)	4.7
	left temporal	4	4.4 (3.1–5.9)	26.1	S25-S31 (10.0–12.4 mm)	11.3
	right temporal	5	7.1 (5.4–9.6)	24.4	S21-S34 (8.4–13.6 mm)	18.9
	parietal	4	5.5 (4.1–6.4)	18.0	S26-S32 (10.4–12.8 mm)	8.9
C	vertex posterior	5	10.6 (8.3–12.6)	14.6	S32-S33 (12.8–13.2 mm)	1.7
	left temporal	4	6.0 (4.6–7.0)	17.1	S25-S30 (10.0–12.0 mm)	7.7
	right temporal	5	8.0 (6.4–12.1)	29.7	S26-S30 (10.4–12.0 mm)	6.0
	parietal	5	20.3 (15.5–26)	19.4	S26-S35 (10.4–14.0 mm)	11.9

When compared to hairs collected from the left temporal, right temporal, and parietal regions, the 5 hairs collected from the vertex posterior region showed a more consistent drug detection, with a smaller relative standard deviation for the drug concentration and peak position than was obtained for the other three regions. The Society of Hair Testing also recommends the vertex posterior region as a hair sampling site (Sachs, 1997). The concentration peak was more consistent in the vertex posterior region, indicating that the vertex posterior area has less variability in terms of hair growth rate. The average linear growth rate of human hair at the vertex posterior regions of the scalp is widely accepted to be approximately 1 cm/month according to the current literature (Bost, 1993). In addition, hair growth occurs in three stages: the anagen (growing), catagen (involution), and telogen (resting) phases (Suzu et al., 1992). Approximately 10%–20% of hair is in the catagen or telogen stage (Salomone et al., 2017). The detection rate for zolpidem was higher in 20 hairs from the vertex posterior region (100%) than from the left temporal (90%) and parietal (95%) regions in the three subjects, in agreement with the previous theory that the proportion of telogen hair is lowest in the area of the vertex posterior (Pragst and Balikova, 2006; Bost, 1993; Kintz et al., 2006).

Few previous studies have examined drug concentrations in hair from different regions of the scalp. Polettini et al. (2012) collected hair from the frontal, anterior vertex, posterior vertex, nape, and temporal regions with clippers to compare the concentrations of MAMP and AMP. Higher concentrations of MAMP and AMP were detected in the nape, temporal, and posterior vertex regions than in the frontal and anterior vertex regions, but we did not observe this pattern in our subjects. To our knowledge, this is the first study to use micro-segmental analysis to investigate drug distribution differences in single hairs collected from different regions of the scalp. The analysis of a single hair can reduce the interference of the blank hair matrix and can better reflect the hair growth rate and growth stage in different scalp regions of the same individual.

## 4 Conclusion

Single hairs collected at 1 day after intake showed a peak of zolpidem in hair at proximal S1 with a concentration of 1.5–2.4 pg/mm and much lower concentrations (< 1 pg/mm) commonly found between S2 and S8. The drug concentration in S1 of hair samples decreased gradually at 3 days, 7 days, and 28 days after drug intake, with the positive band gradually moving toward the tip side, suggesting that the drug in the bloodstream first combines with the hair follicle and is then gradually moved to the hair tip as the hair grows. The zolpidem concentration–hair segment profiles also exhibited a large peak (root side) and a small peak (tip side) for four sampling times in all three test subjects, suggesting that zolpidem incorporation in the hair bulb occurs from the blood, but it also probably enters hair through sweat and sebum. Zolpidem was detected in all hairs from the vertex posterior and right temporal region in all three subjects, but it was not detected in 1 hair from the parietal region or in 2 hairs from the left temporal region. Drug detection was more consistent and showed smaller CVs for drug concentration and peak position when collected from the vertex posterior than from the other three scalp regions, suggesting that the vertex posterior is a suitable sampling region for estimating drug intake.

## Data Availability

The original contributions presented in the study are included in the article/supplementary material, further inquiries can be directed to the corresponding author.

## References

[B1] Bost R. O. (1993). Hair analysis--perspectives and limits of a proposed forensic method of proof: A review. Forensic Sci. Int. 63, 31–42. 10.1016/0379-0738(93)90257-b 8138232

[B2] Cone E. J. (1996). Mechanisms of drug incorporation into hair. Ther. Drug Monit. 18, 438–443. 10.1097/00007691-199608000-00022 8857565

[B3] Cui X. Xiang P. Zhang J. Shi Y. Shen B. Shen M. (2013). Segmental hair analysis after a single dose of zolpidem: Comparison with a previous study. J. Anal. Toxicol. 37 (6), 369–375. 10.1093/jat/bkt035 23657838

[B4] Günther K. N. Johansen S. S. Wicktor P. Banner J. Linnet K. (2018). Segmental analysis of chlorprothixene and desmethylchlorprothixene in postmortem hair. J. Anal. Toxicol. 42, 642–649. 10.1093/jat/bky038 29945160

[B5] Ishida T. Kudo K. Hayashida M. Ikeda N. (2009). Rapid and quantitative screening method for 43 benzodiazepines and their metabolites, zolpidem and zopiclone in human plasma by liquid chromatography/mass spectrometry with a small particle column. J. Chromatogr. B Anal. Technol. Biomed. Life Sci. 877, 2652–2657. 10.1016/j.jchromb.2009.05.008 19501029

[B6] Ji J. J. Yan H. Xiang P. Wang X. Shen M. (2020). The distribution of quetiapine and 7-hydroxyquetiapine in Guinea pig hair roots and shafts after repeated administration: Exploration of the mechanism of drug entry and retention in hair. J. Anal. Toxicol. 45, 1042–1051. 10.1093/jat/bkaa151 33242096

[B7] Kamata T. Shima N. Sasaki K. Matsuta S. Takei S. Katagi M. (2015). Time-course mass spectrometry imaging for depicting drug incorporation into hair. Anal. Chem. 87, 5476–5481. 10.1021/acs.analchem.5b00971 25919888

[B8] Kim M. S. In S. Choi H. Lee S. (2013). Illegal use of benzodiazepines and/or zolpidem proved by hair analysis. J. Forensic Sci. 58, 548–551. 10.1111/1556-4029.12034 23278299

[B9] Kintz P. Villain M. Cirimele V. (2006). Hair analysis for drug detection. Ther. Drug Monit. 28, 442–446. 10.1097/01.ftd.0000211811.27558.b5 16778731

[B10] Kintz P. (2007). Bioanalytical procedures for detection of chemical agents in hair in the case of drug-facilitated crimes. Anal. Bioanal. Chem. 388, 1467–1474. 10.1007/s00216-007-1209-z 17340077

[B11] Kłys M. Rojek S. Bolechała F. (2005). Determination of oxcarbazepine and its metabolites in postmortem blood and hair by means of liquid chromatography with mass detection (HPLC/APCI/MS). J. Chromatogr. B Anal. Technol. Biomed. Life Sci. 825, 38–46. 10.1016/j.jchromb.2005.02.004 16154521

[B12] Kuwayama K. Nariai M. Miyaguchi H. Iwata Y. T. Kanamori T. Tsujikawa K. (2018a). Micro-segmental hair analysis for proving drug-facilitated crimes: Evidence that a victim ingested a sleeping aid, diphenhydramine, on a specific day. Forensic Sci. Int. 288, 23–28. 10.1016/j.forsciint.2018.04.027 29705586

[B13] Kuwayama K. Nariai M. Miyaguchi H. Iwata Y. T. Kanamori T. Tsujikawa K. (2018b). Accurate estimation of drug intake day by microsegmental analysis of a strand of hair by use of internal temporal markers. J. Appl. Lab. Med. 3, 37–47. 10.1373/jalm.2017.025346 33626832

[B14] Kuwayama K. Nariai M. Miyaguchi H. Iwata Y. T. Kanamori T. Tsujikawa K. (2019). Estimation of day of death using micro-segmental hair analysis based on drug use history: A case of lidocaine use as a marker. Int. J. Leg. Med. 133, 117–122. 10.1007/s00414-018-1939-9 30242469

[B15] Kuwayama K. Miyaguchi H. Kanamori T. Tsujikawa K. Yamamuro T. Segawa H. (2021). Development of an improved method to estimate the days of continuous drug ingestion, based on the micro-segmental hair analysis. Drug Test. Anal. 13, 1295–1304. 10.1002/dta.3025 33682351

[B16] Madry M. M. Kraemer T. Baumgartner M. R. (2020). Large scale consumption monitoring of benzodiazepines and z-drugs by hair analysis. J. Pharm. Biomed. Anal. 183, 113151–151. 10.1016/j.jpba.2020.113151 32092690

[B17] Pannatier A. Jenner P. Testa B. Etter J. C. (1978). The skin as a drug-metabolizing organ. Drug Metab. Rev. 8, 319–343. 10.3109/03602537808993791 363387

[B18] Polettini A. Cone E. J. Gorelick D. A. Huestis M. A. (2012). Incorporation of methamphetamine and amphetamine in human hair following controlled oral methamphetamine administration. Anal. Chim. Acta 726, 35–43. 10.1016/j.aca.2012.01.042 22541011 PMC3391534

[B19] Pötsch L. Skopp G. Moeller M. R. (1997). Biochemical approach on the conservation of drug molecules during hair fiber formation. Forensic Sci. Int. 84, 25–35. 10.1016/s0379-0738(96)02045-2 9042707

[B20] Pragst F. Balikova M. A. (2006). State of the art in hair analysis for detection of drug and alcohol abuse. Clin. Chim. Acta 370, 17–49. 10.1016/j.cca.2006.02.019 16624267

[B21] Pragst F. Rothe M. Spiegel K. Sporkert F. (1998). Illegal and therapeutic drug concentrations in hair segments - a timetable of drug exposure? Forensic Sci. Rev. 10, 81–111. 26255716

[B22] Rossi S. Anzillotti L. Castrignanò E. Frison G. Zancanaro F. Chiarotti M. (2014). UHPLC-MS/MS and UHPLC-HRMS identification of zolpidem and zopiclone main urinary metabolites and method development for their toxicological determination. Drug Test. Anal. 6, 226–233. 10.1002/dta.1470 23512850

[B23] Sachs H. (1997). Quality control by the society of hair testing. Forensic Sci. Int. 84, 145–150. 10.1016/s0379-0738(96)02057-9 9042719

[B24] Salomone A. Palamar J. J. Gerace E. Di Corcia D. Vincenti M. (2017). Hair testing for drugs of abuse and new psychoactive substances in a high-risk population. J. Anal. Toxicol. 41, 376–381. 10.1093/jat/bkx020 28334805 PMC5427665

[B25] Schräder J. Rothe M. Pragst F. (2012). Ethyl glucuronide concentrations in beard hair after a single alcohol dose: Evidence for incorporation in hair root. Int. J. Leg. Med. 126, 791–799. 10.1007/s00414-012-0729-z 22773311

[B26] Shima N. Sasaki K. Kamata T. Matsuta S. Wada M. Kakehashi H. (2017). Incorporation of zolpidem into hair and its distribution after a single administration. Drug Metab. Dispos. 45, 286–293. 10.1124/dmd.116.074211 27974380

[B27] Suzu S. Ohtsuki T. Yanai N. Takatsu Z. Motoyoshi K. Takaku F. (1992). Identification of a high molecular weight macrophage colony-stimulating factor as a glycosaminoglycan-containing species. J. Biol. Chem. 267, 4345–4348. 10.1016/s0021-9258(18)42841-4 1531650

[B28] Villain M. Chèze M. Dumestre V. Ludes B. Kintz P. (2004). Hair to document drug-facilitated crimes: Four cases involving bromazepam. J. Anal. Toxicol. 28, 516–519. 10.1093/jat/28.6.516 15516307

[B29] Wang T. Shen B. Wu H. Hu J. Xu H. Shen M. (2018). Disappearance of R/S-methamphetamine and R/S-amphetamine from human scalp hair after discontinuation of methamphetamine abuse. Forensic Sci. Int. 284, 153–160. 10.1016/j.forsciint.2018.01.011 29408724

[B30] Xiang P. Shen M. Drummer O. H. (2015). Review: Drug concentrations in hair and their relevance in drug facilitated crimes. J. Forensic Leg. Med. 36, 126–135. 10.1016/j.jflm.2015.09.009 26454219

[B31] Xu D. Ji J. Xiang P. Yan H. Shen M. (2022). Two DFSA cases involving midazolam clarified by the micro-segmental hair analyses. Forensic Toxicol. 40, 374–382. 10.1007/s11419-022-00621-1 36454413

[B32] Yan H. Xiang P. Shen M. (2021). Current status of hair analysis in forensic toxicology in China. Forensic Sci. Res. 6, 240–249. 10.1080/20961790.2021.1921945 34868718 PMC8635620

